# Associations of C1q/TNF-Related Protein-9 Levels in Serum and Epicardial Adipose Tissue with Coronary Atherosclerosis in Humans

**DOI:** 10.1155/2015/971683

**Published:** 2015-09-17

**Authors:** Jing Wang, Tao Hang, Xun-min Cheng, De-min Li, Qi-gao Zhang, Li-jun Wang, Yong-ping Peng, Jian-bin Gong

**Affiliations:** ^1^Department of Cardiology, Jinling Hospital, Clinical School of Medical College, Nanjing University, Nanjing 210002, China; ^2^Department of Cardiothoracic Surgery, Jinling Hospital, Clinical School of Medical College, Nanjing University, Nanjing 210002, China

## Abstract

*Objective*. To investigate the correlation of CTRP9 with coronary atherosclerosis. *Methods*. Coronary angiography confirmed CAD in 241 patients (62 received CABG) and non-CAD in 121 (55 received valve replacement). *Results*. Serum levels of LDL-C, CRP, TNF-*α*, IL-6, and leptin in CAD patients were significantly higher than those in non-CAD patients (*P* < 0.05), but APN and CTRP9 were lower (*P* < 0.05). Serum levels of CTRP9 and APN were negatively related to BMI, HOMA-IR, TNF-*α*, IL-6, and leptin but positively to HDL-C (*P* < 0.05) in CAD patients. After adjustment of APN, CTRP9 was still related to the above parameters. Serum CTRP9 was a protective factor of CAD (*P* < 0.05). When compared with non-CAD patients, leptin mRNA expression increased dramatically, while CTRP9 mRNA expression reduced markedly in epicardial adipose tissue of CAD patients (*P* < 0.05). The leptin expression and macrophage count in CAD group were significantly higher than in non-CAD group, but CAD patients had a markedly lower CTRP9 expression (*P* < 0.05). *Conclusions*. Circulating and coronary CTRP9 plays an important role in the inflammation and coronary atherosclerosis of CAD patients. Serum CTRP9 is an independent protective factor of CAD.

## 1. Introduction

It has been well demonstrated that adipose tissue is an active endocrine organ and can secrete a variety of bioactive molecules known as adipokines. These adipokines may modulate insulin sensitivity and function in a pro- or anti-inflammation capacity in metabolic dysfunction [1, 2]. Epicardial adipose tissue (EAT) is an unusual visceral fat depot with anatomical and functional contiguity to the myocardium and coronary arteries and can secrete adipokines and directly mediate the occurrence and extent of coronary atherosclerosis. Thus, EAT represents as a novel therapeutic target for obesity-related cardiovascular diseases [3, 4]. Recently, a highly conserved family of adiponectin (APN) paralog known as C1q/TNF-Related Protein (CTRP) is identified. This family has been reported to provide a link between inflammation and metabolism [5, 6]. Of CTRPs, CTRP9 is the closest paralog of APN and has the highest amino acid sequence homology (54%) at the globular domain to APN [6]. However, few studies have been conducted to investigate CTRP9, and available studies focused on animals. Thus, the role of CTRP9 in the pathogenesis of human diseases, especially CAD, is poorly understood. In the present study, CTRP9 and other adipokines were detected in the serum and EAT of CAD patients, and the relationship of circulating and coronary CTRP9 with the coronary atherosclerosis (CA) and its risk factors was further evaluated, which may elucidate whether the role of these factors in CAD is independent of APN.

## 2. Subjects and Methods

### 2.1. Subjects

A total of 362 patients received coronary angiography in the Department of Cardiology of Nanjing Jinling Hospital between December 2012 and December 2014. Of them, 241 patients were diagnosed with CAD (62 received coronary artery bypass grafting (CABG) in the Department of Cardiothoracic Surgery) and 121 with non-CAD (55 underwent valve replacement). Exclusion criteria included acute myocardial infarction, congestive heart failure, cancer, thyroid disease, chronic renal insufficiency, and use of oral corticosteroids. Written informed consent was obtained from each patient before study, and this study was approved by the Ethics Committee of our hospital.

### 2.2. Clinical Data

Clinical information was obtained using a standardized health questionnaire. Body weight (kg) and blood pressure (BP, mmHg) were measured, and body mass index (BMI) was calculated. On admission, blood samples were collected in the morning after an overnight fast (12–14 h). Fasting blood glucose (FBG), total cholesterol (TC), triglyceride (TG), low-density lipoprotein-cholesterol (LDL-C), high-density lipoprotein-cholesterol (HDL-C), C-reactive protein (CRP), and fasting insulin were measured with an automatic biochemical analytical instruments in the Laboratory Center of Nanjing Jinling Hospital. The insulin sensitivity was estimated with the homeostasis model assessment (HOMA) which is based on fasting glucose and insulin concentrations [7]. Samples of adipose tissue were collected during CABG and valve replacement. EAT (0.5 g on average) was collected from the anterior interventricular sulcus. These samples were divided into two parts. One was frozen in liquid nitrogen and stored at −80°C for mRNA extraction, and the other was fixed in paraformaldehyde for immunohistochemistry.

### 2.3. Diagnostic Criteria

Diagnostic criteria for hypertension were as follows: systolic blood pressure was ≥140 mmHg and/or diastolic blood pressure was ≥90 mmHg, or patients had a history of antihypertensive treatment. Diagnostic criteria for diabetes were as follows: FBG was ≥7.0 mmol/L, 2-hour postprandial blood glucose was ≥11.1 mmol/L, or patients were treated with insulin or glucose-lowering drugs.

### 2.4. Percutaneous Coronary Angiography

Conventional coronary angiography was performed according to Judkins' procedures via the femoral artery or radial artery, and at least five projections were employed in each patient. Quantitative coronary angiographic analysis was done by calibration and measurement of coronary dimensions with a digital subtraction angiography system (INNOVA 3100, GE Healthcare, Waukesha, WI, USA) by two experienced interventional cardiologists. CAD was defined as 50% or greater stenosis of a coronary artery.

### 2.5. Quantitative RT-PCR

In brief, 100 mg EAT/TAT was homogenized and total RNA was extracted and purified according to the manufacturer's instructions (Beyotime Institute of Biotechnology). The mRNA purity was determined by measuring the optical density (OD) at 260 nm (A260) and 280 nm (A280). Additionally, the mRNA integrity was approximated by 19S ribosomal bands. Primers for target genes were designed, and RT-PCR was conducted to quantify the mRNA expression of target genes with a 480 real-time PCR system (Roche Applied Science, Indianapolis, IN, USA) following the standard protocol. Primers for leptin, CTRP9, and *β*-actin are showed in Table 1. The fold change in mRNA expression was determined after normalization to *β*-actin mRNA expression.

### 2.6. Immunohistochemistry

EAT was embedded in paraffin and cut into 4 *μ*m sections for hematoxylin and eosin (H&E) staining. Sections were also treated with mouse anti-human leptin monoclonal antibody (Novus Biologicals, USA), rabbit anti-human CTRP9 antibody (Aviscera Bioscience, USA), or mouse anti-human macrophage antibody (Santa Cruz, USA). After visualization, these sections were observed under a microscope and representative images were analyzed with Image-Pro Plus 8.0 software (Media Cybernetics, Cambridge, MA, USA).

### 2.7. ELISA

ELISAs were performed according to the manufacturer's instructions (Nanjing Well-Offer Biotechnology Co. Ltd.). In brief, check-board titration was used to optimize the concentration of coated and capture antibodies. A standard curve was delineated on the basis of OD of standard samples. The concentrations of target proteins were determined according to the standard curve.

### 2.8. Statistical Analysis

Statistical analysis was carried out with SPSS version 19.0 (SPSS Inc., Chicago, IL, USA). Continuous quantitative variables are expressed as mean ± standard deviation (SD) and binary variables as percentages. *χ* ^2^ test was employed for intergroup comparison. Quantitative data were analyzed with independent Student's *t*-test or one-way ANOVA among groups. GraphPad prism 5.0 was used for the correlation analysis and partial correlation analysis for dual-variant data. Multiple logistic regression analysis was used to calculate an adjusted odd ratio (OR) for the occurrence of CAD, and results were presented as OR with a 95% confidence interval (CI). A two-tailed value of *P* < 0.05 was considered statistically significant.

## 3. Results

### 3.1. Characteristics of Subjects at Baseline

In the present study, there were no significant differences in the risk factors of CAD (including age, gender, smoking status, diabetes, hypertension, and BMI) between two groups. Moreover, the serum levels of TC, TG, HDL-C, and FBG between groups were also comparable (*P* > 0.05). However, the serum levels of LDL-C, CRP, TNF-*α*, IL-6, and leptin in CAD patients were markedly higher than those in non-CAD patients (*P* < 0.05), and serum levels of APN and CTRP9 in CAD patients were dramatically lower than those in non-CAD patients (*P* = 0.021 and *P* = 0.005, resp.) (Table 2).

### 3.2. Association of CTRP9 and APN with Cardiovascular Risk Factors

Serum levels of CTRP9 and APN were positively related to BMI, HOMA-IR, TNF-*α*, IL-6, and leptin (*P* < 0.05) but negatively to HDL-C (*P* < 0.05) in CAD patients. In addition, there was a positive relationship between serum CTRP9 and serum APN (*r* = 0.769, *P* < 0.001). After adjustment for APN, CTRP9 was still associated with above parameters, but the extent of its correlation with BMI, HOMA-IR, and HDL-C slightly reduced (*r* = −0.211–−0.207, *P* = 0.035; *r* = −0.249–−0.191, *P* = 0.045; and *r* = 0.240–0.218, *P* = 0.044, resp.) (Table 3).

### 3.3. Multiple Logistic Regression Analysis of Independent Predictors of CAD

Multiple logistic regression analysis showed serum levels of APN and CTRP9 were protective factors of CAD (OR = 0.504, *P* = 0.002 and OR = 0.620, *P* = 0.011, resp.), but serum levels of TNF-*α* and leptin were independent risk factors of CAD (OR = 3.091, *P* = 0.039 and OR = 2.103, *P* = 0.042, resp.). However, serum IL-6 had no influence on CAD (*P* = 0.120) (Table 4).

### 3.4. Inflammatory Cytokines in EAT

**Figure 1:**
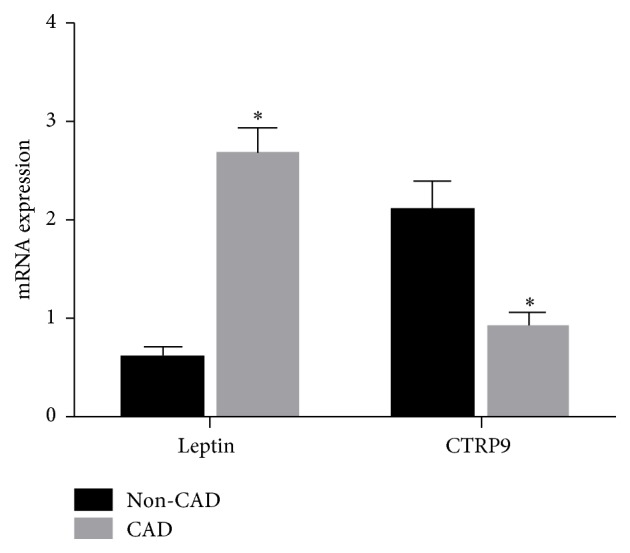
mRNA expressions of leptin and CTRP9 in the EAT. CTRP9: C1q/TNF-Related Protein-9; non-CAD: noncoronary artery disease; CAD: coronary arterial disease; EAT: epicardial adipose tissue volume; ^*∗*^ *P* < 0.05.

**Figure 2:**
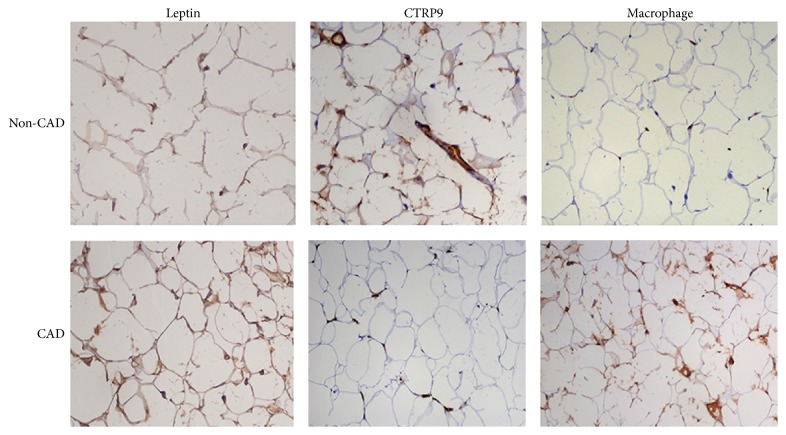
Immunohistochemistry for leptin, CTRP9, and macrophage marker in the EAT. CTRP9: C1q/TNF-Related Protein-9; non-CAD: noncoronary artery disease; CAD: coronary heart disease.

EAT was collected during CABG and valve replacement, and inflammatory cytokines were measured. When compared with non-CAD patients, the leptin mRNA expression in the EAT increased markedly (*P* < 0.05), but CTRP9 mRNA expression in the EAT reduced dramatically (*P* < 0.05, Figure 1) in CAD patients. Immunohistochemistry for leptin and macrophage marker in the EAT showed that leptin expression and macrophage count in CAD patients were significantly higher than those in non-CAD patients, while the CTRP9 expression reduced markedly in CAD patients (Figure 2).

## 4. Discussion

Adipose tissues have been proved to be a metabolically active organ capable of secreting endocrine and paracrine agents that may potentially affect the cardiovascular system and are independent risk factors of coronary arterial disease (CAD). Leptin was identified as the first adipokine in 1994 and is able to promote the progression of diabetes, obesity, and atherosclerosis [8]. On the contrary, APN, a member of CTRP family, has attracted much interest because of its anti-inflammatory/vasculoprotective and anti-ischemic/cardioprotective properties. To date, 15 additional members of the CTRP family have been identified [6]. CTRP9, the closest adiponectin paralog, is predominantly expressed in adipose tissues. CTRP9 has attracted a lot of attention due to its beneficial cardiovascular effects, especially the antiatherosclerotic effect [9–12].

EAT is a heart fat depot and directly contacts with the myocardium and coronary arteries. The pathological increase in EAT and the concomitance of other metabolic and hemodynamic abnormalities turn it into an adverse lipotoxic, prothrombotic, and proinflammatory organ [13–15]. Our previous study showed that pericardial adipose tissue volume was significantly correlated with traditional cardiovascular risk factors, the severity of CA, and the number of stenotic coronary vessels [16]. EAT-derived inflammatory cytokines are also significantly related to the plaque vulnerability in CAD. When compared with non-CAD patients, more macrophages were found, and the expressions of leptin and MMP9 increased markedly in the EAT of CAD patients [17]. Thus, in the present study, the serum and EAT levels of CTRP9 and other proinflammatory cytokines were detected in patients with and without CAD, aiming at elucidating the influence of circulating and coronary CTRP9 on CAD.

Our results showed there were no marked differences in the age, gender, drinking status, smoking status, hypertension, diabetes, and BMI between CAD patients and non-CAD patients. In CAD patients, the serum levels of LDL-C, CRP, TNF-*α*, IL-6, and leptin increased significantly and serum levels of APN and CTRP9 reduced markedly as compared to non-CAD patients. We further correlated serum levels of CTRP9 and APN with risk factors of cardiovascular diseases. Results showed both CTRP9 and APN were protective factors of CAD, negatively related to BMI, HOMA-IR, TNF-*α*, IL-6, and leptin (risk factors) and positively related to HDL-C (a protective factor). Moreover, there was a positive correlation between CTRP9 and APN. These findings suggest that CTRP9 like APN may exert antidiabetic and anti-inflammatory effects and regulate the glucose homeostasis primarily through improving insulin sensitivity.

CTRP9 and adiponectin can form heterotrimers via the globular C1q domain that circulates in the blood [18] and shares the receptor Adipo R1 with adiponectin in the vascular endothelial cells [19] and cardiomyocytes [20]. Therefore, we examined whether the favorable metabolic effects of CTRP9 were adiponectin dependent. After adjustment for APN with partial correlation analysis, CTRP9 was still negatively associated with BMI, HOMA-IR, TNF-*α*, IL-6, and leptin and positively related to HDL although the extent of its relationship with BMI, HOMA-IR, and HDL-C reduced slightly. Multivariate logistic regression analysis revealed that serum CTRP9 was an independent protective factor of CAD (OR = 0.620, *P* = 0.011). This suggests that the antidiabetic, anti-inflammatory, and myocardial protective effects of CTRP9 are independent of APN although the relationship of CTRP9 and APN with risk factors of cardiovascular diseases is comparable and there is a relationship between CTRP9 and APN. In recent 2 studies, similar findings were obtained: serum CTRP9 level was positively associated with favorable glucose or metabolic phenotypes [7] and arterial stiffness in patients with type 2 diabetes [12].

In this study, EAT was collected around the coronary artery during CABG and valve replacement. Results showed that when compared with non-CAD patients, leptin mRNA expression increased markedly but the CTRP9 mRNA expression reduced significantly in EAT of CAD patients. Immunohistochemistry for leptin and macrophage marker in EAT showed that the leptin expression and macrophage count were significantly higher in CAD patients, but the CTRP9 expression was lower in CAD patients as compared to non-CAD patients. CA is influenced by not only the proinflammatory and anti-inflammatory adipokins in the systemic circulation, but also the inflammatory status around the coronary vessels. With the development of CA, the anti-inflammatory and vascular protective effects of CTRP9 are compromised. Our previous study showed that, in rabbit CA model, the whiting epicardial brown adipose tissue was present in the progression of CA, and IL-6 could induce the transformation of epicardial brown adipose tissue into white adipose tissue by activating JAK-STAT3 pathway.

Currently, the specific mechanism underlying the effects of CTRP9 on CA is still unclear. In studies on CTRP9 KO mice [21] and transgenic CTRP9 mice [9], results showed that pharmacologically elevated circulating CTRP9 exerted therapeutic effects on the obesity and obesity-associated insulin resistance. Several studies have indicated that CTRP9 is able to exert protective effects on the cardiovascular system. CTRP9 may induce vasorelaxation in an endothelium-dependent manner, which is mediated by the adiponectin receptor-1/AMPK/endothelial nitric oxide synthase/nitric oxide signaling pathway [19]. CTRP9 downregulation by TNF-*α*-initiated oxidative PPAR*γ* suppression contributes to the exacerbated heart injury in diabetes. The C-terminal globular domain of CTRP9 is able to attenuate the oxidative stress, reduce the myocardial infarct area, and enhance cardiac output in DIO mice after myocardial ischemia/reperfusion injury [22]. CTRP9 overexpression reduces myocardial infarct area and hypoxia-induced apoptosis of cardiac myocytes following myocardial ischemia/reperfusion in a AMPK signaling pathway dependent manner [20] and substantially reduces the neointimal formation by suppressing the vascular smooth muscle cell proliferation and the migration through the cAMP/PKA/ERK pathway [11].

## 5. Limitations

There are some limitations in our study. First, this was a cross-sectional study and whether there is causative association among them is still unclear. Second, the study was conducted in a single center and in Chinese patients, the sample size was small, and whether our results can be generalized to other patients is uncertain. In addition, the mechanisms underlying the protective effects of CTRP9 on the cardiovascular system are required for further investigation. Thus, studies with large sample size and long-term follow-up are needed to uncover the underlying mechanisms of the protective effects of CTRP9.

## 6. Conclusion

Our study for the first time investigated the associations of CTRP9 and EAT with CA in humans. In conclusion, serum CTRP9 decreases significantly and the protein and mRNA expression of CTRP9 in EAT reduced markedly in CAD patients as compared to non-CAD patients. Serum CTRP9 is negatively associated with traditional risk factors of cardiovascular diseases and some inflammatory factors but positively with serum APN and HDL-C. Moreover, the above mentioned relationship between CTRP9 and beneficial metabolic phenotypes is independent of serum adiponectin. This study suggests that circulating and coronary CTRP9 plays an important role in the inflammation of CAD and the CA. Serum CTRP9 is an independent protective factor of CAD.

## Figures and Tables

**Table 1:** Primers used for RT-q PCR.

Genes	Forward (5′-3′)	Reverse (5′-3′)
*β*-actin	TTGTAACCAACTGGGACGATATGG	GATCTTGATCTTCATGGTGCTAGG
Leptin	GATGACACCAAAACCCTCATC	GCCACCACCTCTGTGGAGTAG
CTRP9	ACCATGAGGATCTGGTGGCTTCTG	TGTGTCATCGTCCT CATCAGC

CTRP9: C1q/TNF-Related Protein-9.

**Table 2:** Characteristics of subjects in the present study at baseline (*n* = 362).

	Non-CAD (*n* = 121)	CAD (*n* = 241)	*P* value
Age (years)	61.29 ± 11.47	62.51 ± 10.41	0.734
Male (%)	89 (73.55)	172 (71.37)	0.969
Smoking (%)	50 (41.32)	103 (42.74)	0.864
Drinking (%)	43 (35.54)	97 (40.25)	0.493
DM (%)	33 (27.27)	95 (39.42)	0.072
HBP (%)	77 (63.64)	140 (58.09)	0.430
BMI (kg/m^2^)	24.08 ± 1.62	25.02 ± 3.50	0.239
TC (mmol/L)	3.89 ± 1.06	4.05 ± 0.89	0.492
TG (mmol/L)	1.58 ± 1.37	1.67 ± 0.84	0.727
HDL-C (mmol/L)	1.13 ± 0.22	1.05 ± 0.19	0.114
LDL-C (mmol/L)	1.98 ± 0.61	2.48 ± 0.78	0.009
FBG (mmol/L)	5.61 ± 2.08	6.50 ± 3.35	0.256
HOMA-IR	1.50 ± 1.23	2.69 ± 1.01	0.025
CRP (mg/L)	1.41 ± 1.07	5.81 ± 1.23	0.034
TNF-*α* (pg/mL)	7.31 ± 2.87	8.26 ± 3.71	0.019
IL-6 (pg/mL)	3.69 ± 0.82	4.96 ± 1.35	0.039
Leptin (pg/mL)	7.42 ± 2.69	8.23 ± 3.05	0.020
APN (ng/mL)	10.67 ± 2.74	9.41 ± 1.49	0.021
CTRP9 (pg/mL)	96.14 ± 33.13	83.89 ± 36.18	0.005

DM: diabetes mellitus; HBP: hypertension; BMI: body mass index; TC: total cholesterol; TG: triglyceride; LDL-C: low-density lipoprotein-cholesterol; HDL-C: high-density lipoprotein-cholesterol; FBG: fasting blood glucose; HOMA-IR: homeostasis model of assessment for insulin resistance; CRP: C-reactive protein; TNF-*α*: tumor necrosis factor-*α*; IL-6: interleukin-6; APN: adiponectin; CTRP9: C1q/TNF-Related Protein-9.

**Table 3:** Correlation of CTRP9 and APN with risk factors of cardiovascular diseases.

	CTRP9		APN		Adjusted APN	
	*r*	*P* value	*r*	*P* value	*r*	*P* value
BMI (kg/m^2^)	−0.211	0.032	−0.247	0.020	−0.207	0.035
TC (mmol/L)	−0.073	0.500	−0.029	0.791	−0.048	0.486
TG (mmol/L)	−0.085	0.494	−0.013	0.905	−0.056	0.597
HDL-C (mmol/L)	0.240	0.024	0.390	0.018	0.218	0.044
LDL-C (mmol/L)	−0.069	0.525	−0.070	0.375	−0.036	0.655
HOMA-IR	−0.249	0.032	−0.276	0.022	−0.191	0.045
CRP (mg/L)	−0.059	0.613	−0.170	0.146	−0.068	0.581
TNF-*α* (pg/mL)	−0.484	<0.001	−0.319	<0.001	−0.388	<0.001
IL-6 (pg/mL)	−0.295	<0.001	−0.198	<0.001	−0.228	<0.001
Leptin (pg/mL)	−0.662	<0.001	−0.529	<0.001	−0.803	<0.001
APN (ng/mL)	0.769	<0.001				

BMI: body mass index; TC: total cholesterol; TG: triglyceride; LDL-C: low-density lipoprotein-cholesterol; HDL-C: high-density lipoprotein-cholesterol; HOMA-IR: homeostasis model of assessment for insulin resistance; CRP: C-reactive protein; TNF-*α*: tumor necrosis factor-*α*; IL-6: interleukin-6; APN: adiponectin; CTRP9: C1q/TNF-Related Protein-9.

**Table 4:** Multiple logistic regression analysis of independent predictors of coronary arterial disease.

	*P* value	OR	95% CI	
			Lower	Upper
APN	0.002	0.504	0.303	0.720
CTRP9	0.011	0.620	0.482	0.838
Leptin	0.042	2.103	1.003	3.813
TNF-*α*	0.039	3.091	1.204	4.186
IL-6	0.120	1.185	0.957	1.467

TNF-*α*: tumor necrosis factor-*α*; IL-6: interleukin-6; APN: adiponectin; CTRP9: C1q/TNF-Related Protein-9.
